# Integrating Multi-View Features via Deep Generalized Canonical Correlation Analysis for Single-Cell Clustering

**DOI:** 10.3390/ijms27135819

**Published:** 2026-06-27

**Authors:** Wenhao Liu, Wei Zhang, Xiaoying Zheng, Yuanyuan Li

**Affiliations:** School of Mathematics and Physics, Wuhan Institute of Technology, Wuhan 430205, China; hzhu1527@gmail.com (W.L.); wzhang_math@whu.edu.cn (W.Z.)

**Keywords:** multi-view clustering, unsupervised clustering, subspace learning, single-cell RNA sequencing, deep generalized canonical correlation analysis

## Abstract

Single-cell RNA sequencing data are characterized by high dimensionality, sparsity, and strong nonlinearity, hindering conventional single-view clustering methods from capturing linear and nonlinear feature subspaces simultaneously. Features from distinct dimensionality reduction approaches are inherently complementary: PCA (Principal Component Analysis) preserves global linear structures, UMAP (Uniform Manifold Approximation and Projection) maintains topology and local neighborhoods, and PHATE (Potential of Heat-diffusion for Affinity-based Trajectory Embedding) depicts gradual transitions in cell differentiation. To fuse these complementary sources, we adopt an inter-view correlation maximization paradigm. Canonical Correlation Analysis (CCA) integrates two views by maximizing projection correlation but is limited to pairwise scenarios. We extend it to Generalized Canonical Correlation Analysis (GCCA) for multi-view alignment and introduce a deep autoencoder to construct the DeepGCCA (Deep Generalized Canonical Correlation Analysis) framework. This method generates three views via PCA, UMAP, and PHATE, extracts nonlinear latent features with the autoencoder, projects multi-view representations into a unified subspace under weighted GCCA constraints, and performs K-means clustering. Experiments on the two simulated and three real single-cell datasets evaluated in this study show that DeepGCCA demonstrates competitive performance against all single-view baselines and performs favorably compared to several widely adopted methods. Moreover, downstream marker gene analysis supports the biological interpretability of the resulting clusters within these datasets. Within the scope of this benchmark, DeepGCCA provides a valuable reference for high-precision clustering of single-cell transcriptomic data, offering practical insights into multi-view integration and biological interpretability.

## 1. Introduction

With the rapid advancement of single-cell sequencing technologies, single-cell RNA-seq (scRNA-seq) enables the elucidation of gene expression profiles at the single-cell level, laying a high-resolution foundation for data analysis in life sciences, medicine, and related fields [1,2]. scRNA-seq data are characterized by high dimensionality, sparsity, and substantial noise [3,4]. Furthermore, cell states in single-cell data generally fall into two major categories: continuous transition patterns (e.g., developmental trajectories and cell differentiation) [5], in which gene expression shifts gradually across cells with blurred subpopulation boundaries, hindering conventional clustering methods from identifying distinct cell clusters; and discrete patterns (e.g., diverse immune cell subsets), which feature prominent inter-group heterogeneity yet are plagued by high-dimensional sparsity and noise that obscure biological signals and induce clustering bias [6]. These two inherent data characteristics pose fundamentally different challenges to feature learning and clustering algorithm design. Effectively extracting features from such complex data and achieving accurate cell clustering remain one of the core challenges in single-cell bioinformatics analysis. The accuracy of clustering results directly affects subsequent research tasks, including cell-type annotation and gene regulatory network analysis [7].

Cluster analysis, a core unsupervised learning method, is a pivotal tool for interpreting single-cell data [8]. Current single-cell clustering methods include not only traditional K-means [9] and hierarchical clustering [10], but also probabilistic statistical approaches (e.g., Gaussian mixture models [11]), graph-based machine learning pipelines (e.g., spectral clustering [12], Louvain [13], Leiden [14]), and deep clustering algorithms (e.g., DEC (Deep Embedding Clustering) [15]). Nevertheless, most existing methods depend on single-view feature representations: they either directly adopt raw gene expression profiles or leverage a single dimensionality reduction technique (e.g., PCA (Principal Component Analysis) [16] or UMAP (Uniform Manifold Approximation and Projection) [17]) for feature extraction, before implementing downstream clustering algorithms such as K-means or hierarchical clustering. Even mainstream analytical workflows like Seurat [18] perform clustering mainly based on single-view embeddings derived from PCA. However, single-view clustering methods can only capture certain aspects of data characteristics, with the following limitations: first, a single dimensionality reduction method struggles to capture all latent features of single-cell data, potentially leading to the loss of feature information; second, the nonlinear structure of high-dimensional single-cell data cannot be adequately represented by a single linear or nonlinear dimensionality reduction method; third, single-view features have poor robustness to data noise, often resulting in biased clustering results [19].

Multi-view clustering constructs comprehensive, accurate data representations by integrating complementary and consistent information across multiple perspectives, effectively addressing the inherent limitations of single-view clustering and emerging as a promising research direction in single-cell data analysis [19,20]. In recent years, extensive multi-view learning methods have been developed for computer vision and multimedia tasks, including multi-view subspace clustering (e.g., Latent Multi-View Subspace Clustering, LMSC [21]), multi-view graph learning (e.g., Adaptive Multi-View Graph Learning, AMGL [22]), and multi-view autoencoder-based frameworks (e.g., Joint Deep Multi-View Learning for Image Clustering [23]). Nevertheless, their practical applications to single-cell transcriptomic data remain underexplored [24]. Furthermore, most existing multi-view strategies integrate distinct views via straightforward concatenation or weighted averaging, which cannot capture deep cross-view correlations.

The core pipeline of multi-view clustering consists of two steps: generating diverse feature representations from raw data, and fusing these multi-view features via dedicated strategies to complete clustering. For single-cell data analysis, multi-view representations are generally derived from diverse dimensionality reduction algorithms, each capturing biological patterns from unique perspectives. PCA specializes in linear feature extraction [16], UMAP [17] preserves global topological architecture and local neighborhood relationships, and PHATE effectively characterizes gradual transition patterns during cell differentiation [25]. The integration of these heterogeneous characteristics enables a more holistic depiction of the complex feature distribution of single-cell profiles.

Canonical Correlation Analysis (CCA) [26] is a classic subspace learning algorithm that realizes feature fusion by maximizing the correlation between two projected views, exhibiting notable superiority in dimensionality reduction and cross-modal integration [27,28]. However, conventional CCA is restricted to two-view scenarios and assumes linear feature relationships. To tackle these drawbacks, Generalized Canonical Correlation Analysis (GCCA) extends CCA to multi-view tasks [29,30], while deep learning empowers the model to fit the nonlinear inherent structures of single-cell data [28]. Combined, these advantages motivate the development of our DeepGCCA (Deep Generalized Canonical Correlation Analysis) framework for adaptive multi-view feature fusion in single-cell clustering.

**Figure 1:**
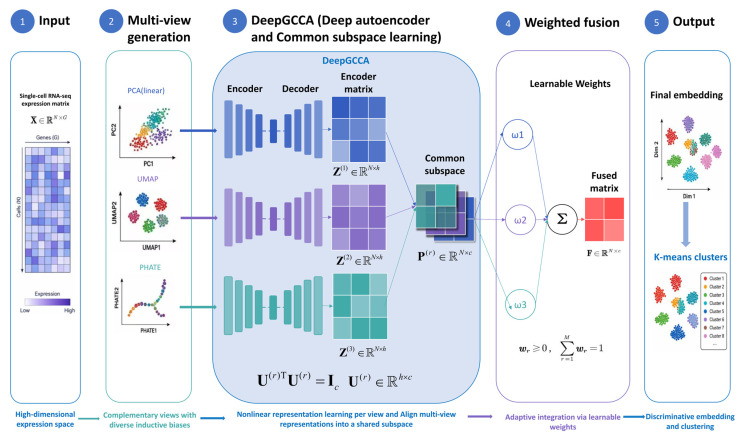
Overview of the DeepGCCA (Deep Generalized Canonical Correlation Analysis) framework for multi-view single-cell clustering.

Therefore, by leveraging the unique characteristics of scRNA-seq data, this study systematically addresses the limitations of traditional single-view methods through multi-view feature generation, public subspace mapping via deep generalized canonical correlation analysis (DeepGCCA), intelligent weighted fusion, and optimized clustering. It provides a high-precision and robust technical solution for cell population classification and cell-type identification in single-cell bioinformatics. The overall architecture of the proposed DeepGCCA framework is illustrated in Figure 1.

## 2. Results

To validate the effectiveness of the proposed DeepGCCA multi-view clustering framework, we conducted systematic experiments on two simulated datasets (Sim1 and Sim2) [31] and three real single-cell RNA sequencing datasets (Mouse Pancreas [32], CellLines [33], and GSE115469 [34]); detailed descriptions of each dataset are provided in Table 1. All datasets were preprocessed identically and exhibited no batch effects, with ground-truth labels used solely for evaluation and K-value determination. The adjusted Rand index (ARI) [35] and normalized mutual information (NMI) [36] were adopted as the key evaluation metrics to quantify clustering performance. The experimental design is structured as follows: first, we compare the proposed method with several state-of-the-art multi-view clustering approaches; second, we visualize the feature representations learned by each method to intuitively assess their separability; third, we perform an ablation experiment to dissect the contribution of each loss component in the framework; fourth, we demonstrate the superiority of multi-view fusion over single-view clustering methods; and finally, we provide a convergence analysis to verify the stability of the proposed model during training.

### 2.1. Comparison with Five Methods

To evaluate the clustering performance of DeepGCCA, we compared it with five representative methods: traditional clustering algorithms (K-means [9], spectral clustering [12]), multi-view approaches specifically designed for single-cell data (scMDC (Clustering of single-cell multi-omics data with a multimodal deep learning method) [37], scMMN (Multi-level multi-view network based on structural contrastive learning for scRNA-seq data clustering) [38]), and the widely used single-cell analysis pipeline Seurat [18]. Given the stochastic nature of several components involved (e.g., UMAP, neural network initialization, and K-means), all experiments were repeated using 20 fixed random seeds, and we report mean ± standard deviation for the Adjusted Rand Index (ARI) and Normalized Mutual Information (NMI) across all methods in Table 2 and Table 3. To assess statistical significance, we performed paired Wilcoxon signed-rank tests comparing DeepGCCA against the strongest baseline (in terms of mean ARI/NMI) on each dataset. DeepGCCA performs at least comparably to the strongest baseline in most settings and significantly outperforms it on the Mouse dataset (*p* < 0.05). On datasets where DeepGCCA is not strictly better (Sim1, CellLines, GSE115469), the differences are not statistically significant (*p* > 0.05), confirming that observed improvements are not mere stochastic artifacts. For Sim2, all methods reached perfect scores across all seeds, precluding meaningful comparison. In addition to the mean performance, we also report the best achievable values for DeepGCCA in the tables to reflect its upper-bound potential under favorable initialization. Complete standard deviations are provided in Tables S1 and S2 of the Supplementary Materials.

In terms of the ARI of Table 2, DeepGCCA achieved the highest mean scores on Sim1 (0.719), Sim2 (1.000, tied), and Mouse (0.613), demonstrating its superior integration capability in these scenarios. On CellLines [33], DeepGCCA (0.650) ranked behind Seurat (0.789), spectral clustering (0.663), and scMDC (0.657), though the gaps among the latter three were within 1%; on GSE115469, it attained 0.562, second only to Seurat (0.601), while substantially outperforming the remaining baselines. For NMI (Table 3), DeepGCCA achieved the highest mean scores on Sim1 (0.748), Sim2 (1.000, tied), Mouse (0.605), and GSE115469 (0.744), and ranked third on CellLines (0.770) behind Seurat (0.836) and scMDC (0.779). Beyond accuracy, we also assessed computational efficiency; detailed runtime and memory usage are provided in the Supplementary Materials (Tables S3–S5). DeepGCCA exhibited moderate computational cost, which did not increase disproportionately with cell count, making it acceptable for most real-world applications.

### 2.2. Visualization of Feature Representations

In addition to quantitative metric evaluation, visual assessment was further conducted to compare clustering separability and structural preservation of low-dimensional feature representations learned by different methods. Two-dimensional embeddings were generated for all datasets across competing approaches. Dimensionality-reduced visualization was first performed on two simulated datasets (Sim1 and Sim2) to evaluate the capacity of each method to recover predefined continuous and discrete cluster structures under controlled conditions. Identical visualization workflows were then applied to three real-world datasets (Mouse Pancreas [32], CellLines [33], and GSE115469 [34]) to assess model robustness amid complex biological noise.

Comparisons covered traditional clustering baselines (K-means, spectral clustering), the mainstream single-cell analytical workflow Seurat, representative multi-view algorithms (scMDC, scMMN), and the proposed DeepGCCA. Distinct from simulated data, real scRNA-seq profiles inherently contain technical noise, batch effects, and biological heterogeneity. Accordingly, low-dimensional embeddings derived from practical data better reveal intrinsic methodological differences in global structural retention, local neighborhood conservation, and inter-cluster discrimination. Uniform preprocessing pipelines and standardized dimensionality reduction parameters were adopted for all visualization procedures to guarantee consistent comparability.

**Figure 2:**
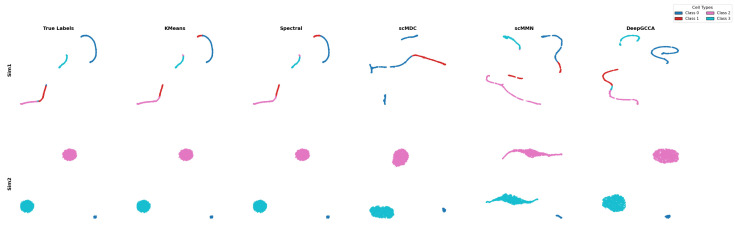
Low-dimensional embeddings of simulated datasets (Sim1 and Sim2) under different clustering methods.

Figure 2 presents comparisons of two simulated datasets (Sim1 and Sim2), along with their two-dimensional embeddings and clustering coloring results across different methods. “True Labels” denote actual categories, while the remaining columns correspond to K-means, spectral clustering, scMDC, scMMN, and DeepGCCA, respectively. This Figure is designed to visually assess the ability of each method to recover predefined population structures, with a focus on category consistency along differentiation branches and the alignment of local mixing patterns at branch junctions with actual experimental setups.

In Sim1, true labels exhibit three primary differentiation trajectories: the upper two branches are dominated by a single category each (blue and light-blue branches), while the lower branch consists primarily of red and pink cells, with a small number of blue and light-blue cells present at branch junctions/transitions. Compared with true labels, K-means and spectral clustering show more prominent category crossings in the upper branches (e.g., red cells mixed into the blue branch and pink cells mixed into the light-blue branch), while failing to replicate the observed mixture of blue and light-blue cells in corresponding regions of the lower branch. This results in inconsistencies between the “mixing locations” and the actual differentiation structure. For this dataset, scMDC generates only two dominant category results (primarily red and blue), failing to adequately distinguish the fine-grained category structure of Sim1. scMMN also exhibits category crossings in the upper branches; additionally, the red and pink trajectories in the lower branch are fragmented, and the connectivity with some blue and light-blue cells does not reflect the true differentiation pattern. In contrast, DeepGCCA reconstructs the Sim1 structure more closely to true labels: the upper branches show better category consistency; the lower branch forms continuous trajectories dominated by red and pink cells, while retaining a small mixture of blue and light-blue cells at junctions, which aligns more closely with true labels. These results demonstrate that DeepGCCA more effectively maintains both category consistency within branches and local structure in transitional regions under continuous differentiation scenarios, yielding embedding and clustering performances more consistent with real-world conditions.

In Sim2 (discrete cluster scenario), true labels exhibit two nearly circular, well-separated primary clusters accompanied by a few outliers. Comparative visualization shows that scMMN significantly stretches the upper primary cluster into an elongated shape, while scMDC also demonstrates a tendency of circular-to-band deformation, with localized fragmentation of small clusters—reflecting inadequate preservation of cluster morphology and boundaries in this discrete scenario. In contrast, K-means, spectral clustering, and DeepGCCA largely replicate the true distribution: primary clusters maintain a compact, nearly circular isotropic structure with clear spacing and no abnormal connections. Among these, DeepGCCA exhibits the highest consistency with true labels in terms of intra-cluster density uniformity, boundary smoothness, and more stable outlier handling. These results indicate that most methods successfully reconstruct primary clusters under typical discrete structures, with DeepGCCA demonstrating superior robustness in both morphology preservation and separation accuracy.

**Figure 3:**
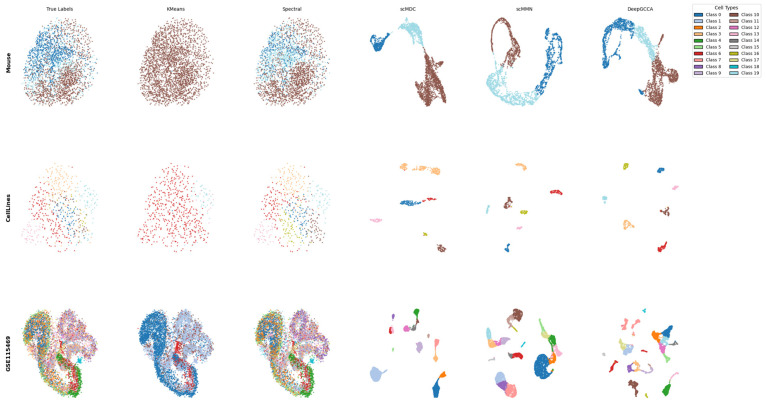
Low-dimensional embeddings of real single-cell datasets (Mouse Pancreas [32], CellLines, and GSE115469 [34]) under different clustering methods.

Figure 3 presents visualization results of three real scRNA-seq datasets (Mouse Pancreas [32], CellLines [33], GSE115469 [34]) in a unified two-dimensional embedding space, colored by clustering labels generated by different methods (K-means, spectral clustering, scMDC, scMMN, and DeepGCCA; the first column displays true labels). Owing to inherent technical noise, batch effects, and biological heterogeneity in real data, true labels often exhibit varying degrees of overlap in the two-dimensional space. This figure is therefore intended to compare the ability of each method to resolve population structures under complex conditions, including the absence of significant under-segmentation, restoration of the expected number of categories, and clarity of cluster boundaries consistent with the spatial distribution trends of true labels.

In the Mouse Pancreas [32] dataset, true labels show some overlap in the embedding space with relatively blurred category boundaries. K-means exhibits a pronounced under-segmentation tendency, clustering the vast majority of cells into a single cluster and failing to reflect the true multi-type architecture. Spectral clustering can distinguish cell populations to a certain extent but still presents significant mixing. In contrast, scMDC, scMMN, and DeepGCCA all generate clearer partitioning structures. Among these, scMDC demonstrates more distinct cluster boundaries and superior spatial separation on this dataset, while DeepGCCA also yields coherent cluster structures with reduced large-scale mixing—indicating that multi-view/deep representation enhances population separability for this dataset.

In the CellLines dataset [33], true labels indicate that multiple cell lines still overlap in the low-dimensional space, and traditional methods have limited capacity to resolve multi-class structures. K-means tends to cluster samples into a few clusters, failing to effectively reconstruct the composition of multiple cell lines. Spectral clustering shows improvement over K-means but remains suboptimal in its correspondence with true labels. Both scMDC and scMMN significantly enhance inter-cluster separation yet have distinct limitations: scMDC fails to fully recover all seven cell types in this dataset (only six major populations are visualized), while scMMN produces a clearer cluster distribution with observable spatial overlap between some clusters. In comparison, DeepGCCA generates more compact clusters with clearer boundaries and higher overall separation, providing a more robust structural foundation for subsequent cluster-based differential expression analysis and cell line annotation.

In the GSE115469 dataset (comprising 20 categories) [34], true labels exhibit highly heterogeneous and complex topological structures in two-dimensional embeddings, reflecting the significant heterogeneity of large-scale real tissue samples. Both K-means and spectral clustering struggle to directly reconstruct the fine-grained category structure: K-means demonstrates a pronounced cluster merging tendency, while spectral clustering identifies more clusters but still produces markedly mixed and unstable partitions. In contrast, scMDC, scMMN, and DeepGCCA significantly improve cluster separation and form multi-cluster structures; however, scMDC fails to reconstruct all 20 categories (approximately 15 major clusters are visualized), indicating limited capacity in identifying highly complex, fine-grained clusters. DeepGCCA generates a more robust and spatially distinct cluster structure across the dataset, with clearer inter-cluster boundaries in multiple regions, demonstrating superior structural resolution capabilities in highly heterogeneous real-world scenarios.

Overall, DeepGCCA provides robust and interpretable representations across both simulated and real scRNA-seq datasets. In the Sim1 continuous simulation scenario with differentiation branches and transitional regions, DeepGCCA most faithfully recovers the predefined branching architecture and the local mixing patterns near transitions, supporting its advantage in modeling complex, non-linear structures. In the Sim2 discrete simulation scenario, most methods can reproduce the ground-truth clusters, whereas scMDC and scMMN induce evident shape distortions (e.g., circular clusters stretched into elongated bands); DeepGCCA shows the highest agreement with true labels in terms of cluster morphology preservation, intra-cluster compactness/density uniformity, and inter-cluster separation. Consistent with these simulation results, across the three real datasets, traditional methods (particularly K-means) are more prone to under-segmentation or category merging, while multi-view/deep approaches generally improve cluster separability and interpretability. Notably, DeepGCCA yields clearer, more coherent, and more separable cluster structures across datasets of varying scales and complexities, with superior category recovery and structural stability in challenging real-world settings such as CellLines [33] and GSE115469 [34]. These qualitative observations align with quantitative metrics (ARI/NMI) and suggest that DeepGCCA provides a reliable clustering foundation for downstream cell-type annotation and functional analyses.

### 2.3. Ablation Study of Loss Components in DeepGCCA

To quantitatively evaluate the impact of the three components in the DeepGCCA total loss function—namely reconstruction loss (Recon), GCCA-related loss (GCCA), and entropy regularization (Entropy)—on clustering performance, we conducted ablation experiments on the Mouse and Sim1 datasets. Specifically, we fixed the weights of the other loss terms at their optimal values while varying only the weight coefficient of the target component (within the set {0, 0.01, 0.1, 1, 5}), and recorded the ARI and NMI scores of the clustering results. The experimental results on the Mouse and Sim1 datasets are summarized in Table 4 and Table S6 of the Supplementary Materials, respectively. The detailed observations are as follows.

As the weight in the Reconstruction Loss increased from 0 to 0.01, the ARI slightly decreased from 0.6581 ± 0.0459 to 0.6548 ± 0.0503, while the NMI marginally rose from 0.7045 ± 0.0680 to 0.7112 ± 0.0541. At a weight of 0.1, both metrics dropped noticeably to their lowest points (ARI = 0.6184 ± 0.0885, NMI = 0.6359 ± 0.1336). However, when the weight was further raised to 1, the performance recovered to the best values within this group (ARI = 0.6812 ± 0.0661, NMI = 0.7341 ± 0.0730). At a weight of 5, the metrics remained essentially stable and comparable to those at weight = 1 (ARI = 0.6817 ± 0.0649, NMI = 0.7290 ± 0.0687). These findings indicate that, apart from a notable dip at the intermediate weight of 0.1, the reconstruction loss generally maintains moderate to optimal performance within the 0–0.01 and 1–5 ranges, with the peak occurring at a weight of 1. GCCA-related Loss: For the GCCA loss, the best performance was observed at a weight of 0.01, with ARI = 0.6812 ± 0.0661 and NMI = 0.7341 ± 0.0730. Increasing the weight to 0.1 led to a clear decline (ARI = 0.6619 ± 0.0842, NMI = 0.6970 ± 0.1071). A further increase to 1 and 5 caused continued and more pronounced degradation, with the lowest values recorded at weight = 5 (ARI = 0.6070 ± 0.0711, NMI = 0.6235 ± 0.1141). Notably, even the absence of GCCA regularization (weight = 0) yielded relatively moderate results (ARI = 0.6449 ± 0.0768, NMI = 0.6632 ± 0.1233), which were still superior to those at weights of 1 and 5. Overall, the GCCA loss is sensitive to over-weighting; larger weights (≥1) consistently impair clustering performance, whereas a small or zero weight is less detrimental. Entropy Regularization: For entropy regularization, the optimal values were attained at a weight of 0.1 (ARI = 0.6812 ± 0.0661, NMI = 0.7341 ± 0.0730). When the weight was set to 0, 0.01, or 1, the performance remained relatively similar, with ARI ranging from 0.6643 to 0.6695 and NMI ranging from 0.6974 to 0.7141. At the largest tested weight (5), however, both metrics decreased to their minimum in this group (ARI = 0.6380 ± 0.0867, NMI = 0.6566 ± 0.1230). These results suggest that entropy regularization is generally robust across a broad range (0–1), but excessively high weights can still cause a non-negligible drop in performance. Based strictly on the tabulated results, the optimal weight for the reconstruction loss is 1, for the GCCA loss is 0.01, and for entropy regularization is 0.1, as each achieves the highest ARI and NMI within its respective category. Importantly, the three loss terms exhibit distinct sensitivity patterns: reconstruction loss shows a bipolar stable behavior (except at 0.1), GCCA loss prefers small weights and deteriorates with large ones, while entropy regularization is broadly stable across 0–1 but suffers at weight = 5. These observations provide direct empirical guidance for selecting appropriate weight configurations in practical applications of the proposed model.

**Figure 4:**
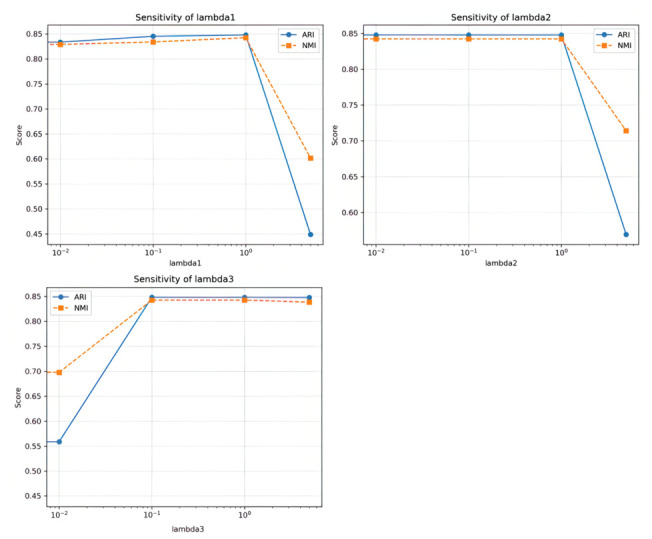
Impact of varying loss term weights on ARI and NMI.

**Figure 5:**
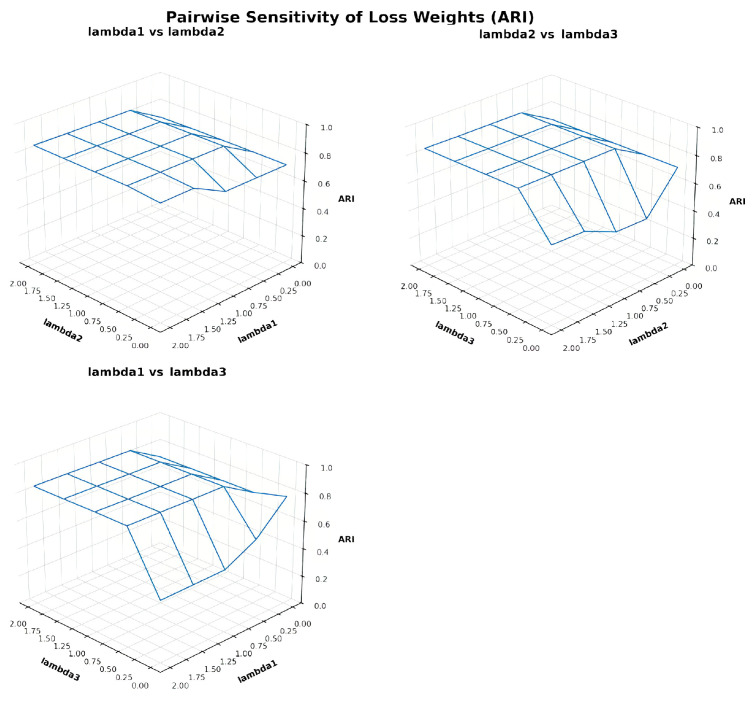
Joint sensitivity analysis of two loss components on ARI.

Figure 4 further visualizes this trend: both the Recon and GCCA curves exhibit performance drop when weights are either excessively small or large, maintaining optimal performance only within a moderate weight range (0.01–1), whereas the Entropy curve shows a severe performance collapse in the absence of regularization, with performance fully restored upon its introduction. To further investigate the interactive effects of these loss weights on clustering performance, Figure 5 shows the contour distribution of ARI under different weight combinations.

As shown in Figure 5, during the joint sensitivity analysis of Recon and GCCA (λ_1_ vs. λ_2_), ARI values remain low regardless of whether λ_1_ or λ_2_ is zero, forming two slopes that converge toward a low valley. For the joint evaluation of Recon and Entropy (λ_1_ vs. λ_3_), the contour map exhibits a steep threshold pattern: when λ_3_ < 0.5, the ARI remains in the low region (<0.6) irrespective of λ_1_; once λ_3_ ≥ 0.5, the ARI increases sharply, forming a clear “cliff-platform” structure. This observation suggests that entropy regularization requires a critical activation threshold. In the joint sensitivity analysis of GCCA and Entropy (λ_2_ vs. λ_3_), the threshold phenomenon reappears: the ARI remains low when λ_3_ < 0.5, whereas for λ_3_ ≥ 0.5, the ARI is mainly governed by λ_2_. In summary, within the weight range of (0–2), entropy regularization represents the critical threshold for achieving high clustering performance, and Recon and GCCA must act synergistically within a moderate weight range.

### 2.4. Comparison of Single-View Versus Multi-View Fusion

**Figure 6:**
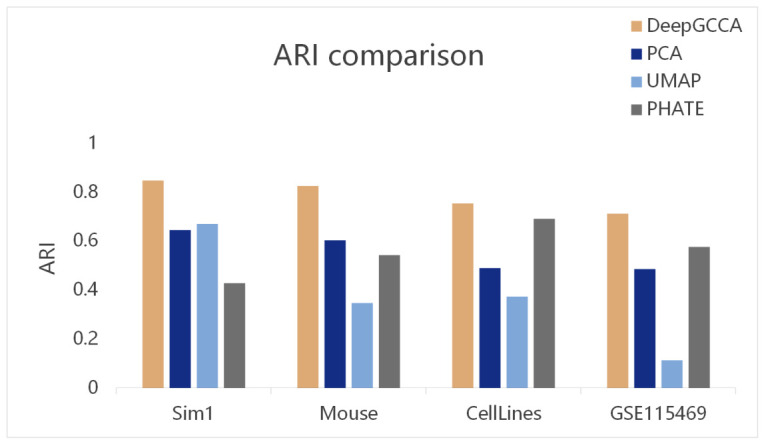
ARI comparison between DeepGCCA and single-view embeddings across datasets.

**Figure 7:**
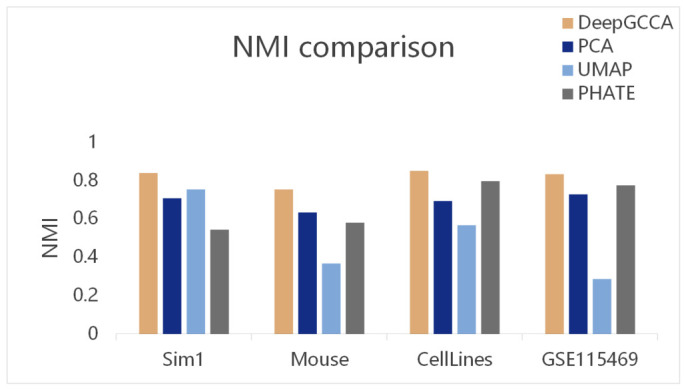
NMI comparison between DeepGCCA and single-view embeddings across datasets.

To evaluate the advantages of the multi-view fusion strategy [39,40] over single-view methods, we compared DeepGCCA with three single-view clustering approaches (PCA, UMAP, and PHATE). All methods used K-means as the base clustering algorithm, and ARI and NMI scores were computed on all five datasets. The results are presented in Figure 6 and Figure 7. ARI comparison between DeepGCCA and single-view methods (PCA, UMAP, PHATE) across four datasets is shown.

The ARI results in Figure 6 demonstrate that DeepGCCA outperforms all single-view methods across all datasets. On the Sim1 dataset, DeepGCCA achieved an ARI of 0.8480, representing improvements of 31.8%, 26.9%, and 98.4% compared to PCA (0.6436), UMAP (0.6685), and PHATE (0.4274), respectively. On the Mouse Pancreas dataset [32], performance variations among single-view methods were significant: UMAP achieved an ARI of only 0.3467, whereas DeepGCCA reached 0.8260—a 37.2% improvement over the optimal single-view method PCA (0.6021). On the CellLines dataset [33], DeepGCCA (0.7547) also significantly outperformed PCA (0.4887), UMAP (0.3726), and PHATE (0.6921). For the GSE115469 dataset [34], DeepGCCA also achieved the highest ARI (0.7133) and NMI (0.8362), outperforming the optimal single-view baseline method PCA (ARI: 0.4847; NMI: 0.7308), with an ARI improvement of 47.2% and an NMI improvement of 14.4%. This further demonstrates the advantages of multi-view fusion on larger-scale real-world datasets.

The NMI results in Figure 7 align with this trend: DeepGCCA maintained its leading performance across all datasets, reaffirming the effectiveness of the multi-view fusion strategy. Notably, on Sim2 all evaluated methods yielded essentially identical, saturated scores (ARI/NMI ≈ 1.0). To avoid redundant visualization and improve readability, the Sim2 results are not included in Figure 6 and Figure 7; the exact values are provided in the corresponding tables.

### 2.5. Convergence of DeepGCCA

**Figure 8:**
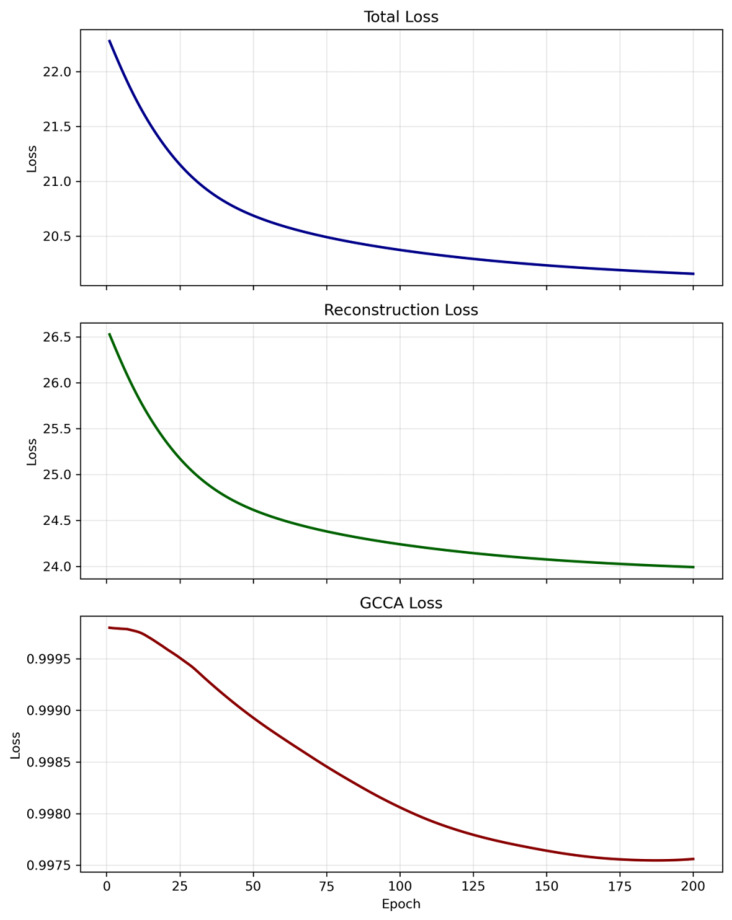
Training convergence curves of total loss, reconstruction loss, and GCCA loss.

To verify the stability and effectiveness of the DeepGCCA model during training, we monitored the total loss, reconstruction loss, and GCCA loss throughout the training process with respect to the number of epochs. The convergence curves are displayed in Figure 8 below.

To evaluate the training stability and efficacy of DeepGCCA, convergence curves of total loss, reconstruction loss, and GCCA loss were monitored across training epochs (Figure 8). All three curves present a continuous, smooth downward trend without obvious oscillations or rebounds, which reflects stable optimization dynamics, controllable training behavior, and steady objective function convergence. The total loss declines rapidly in the early training stage, falling sharply from roughly 22.3 to 20.7 within the first 20–50 epochs, followed by gradual decay and final stabilization near 20.15. Such variation suggests that major feature representations contributing to model optimization are captured at an early stage, while later training is dominated by subtle parameter fine-tuning with marginal performance gains, which conforms to the typical “fast initial decay and slow later convergence” pattern of deep learning frameworks. Among different loss components, reconstruction loss achieves the most remarkable reduction and the fastest convergence. This value drops rapidly from an initial 26.5 and gradually stabilizes at approximately 24.0. This trend verifies that the multi-view autoencoder efficiently captures dominant expression patterns and low-order structural information across distinct views, thereby minimizing reconstruction error at an early stage. After primary structural information is fully learned, residual errors are mainly attributed to inherent sparsity, noise, and view-specific nonlinear characteristics of single-cell data, which slows down the later decay of reconstruction loss.

In comparison, GCCA loss shows the mildest variation and the slowest descending rate, gradually decreasing from 0.9997 to 0.9975 and reaching a plateau after 150 epochs. Cross-view correlation alignment serves as a refined optimization constraint, whose effective improvement relies on stable latent representations generated by individual view encoders, thus leading to slow and steady convergence. Slight fluctuations in the late GCCA curve further confirm that multi-view feature alignment reaches a stable equilibrium in the final training stage. Overall, the loss curves validate the robust convergence of DeepGCCA within 200 epochs. Rapid early optimization is driven by reconstruction-related constraints, while GCCA loss further promotes consistent cross-view alignment in later iterations, jointly facilitating stable total loss convergence. This rational convergence behavior guarantees reliable model optimization and high-quality fused representations for downstream clustering tasks.

### 2.6. Downstream Analysis

**Figure 9:**
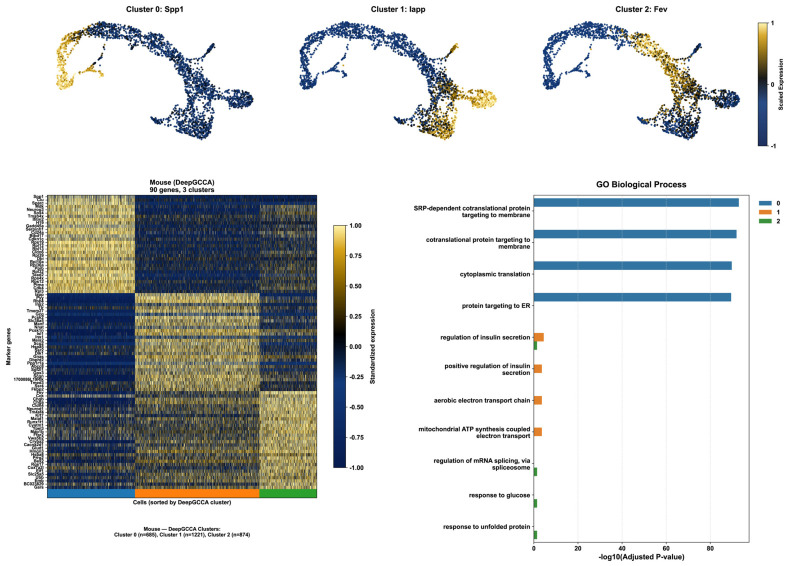
Cell-types heatmap and gene enrichment analysis of the Mouse dataset [32]. Top panels: UMAP projections showing the expression distribution of key marker genes (*Spp1*, *Iapp*, *Fev*) across three distinct cell clusters. Heatmap (**left**): Standardized expression of 90 marker genes across the identified cell clusters, highlighting distinct transcriptional modules. GO enrichment (**right**): Top biological processes enriched in each cluster, with bar lengths indicating the statistical significance (−log10(adjusted *p*-value)).

**Figure 10:**
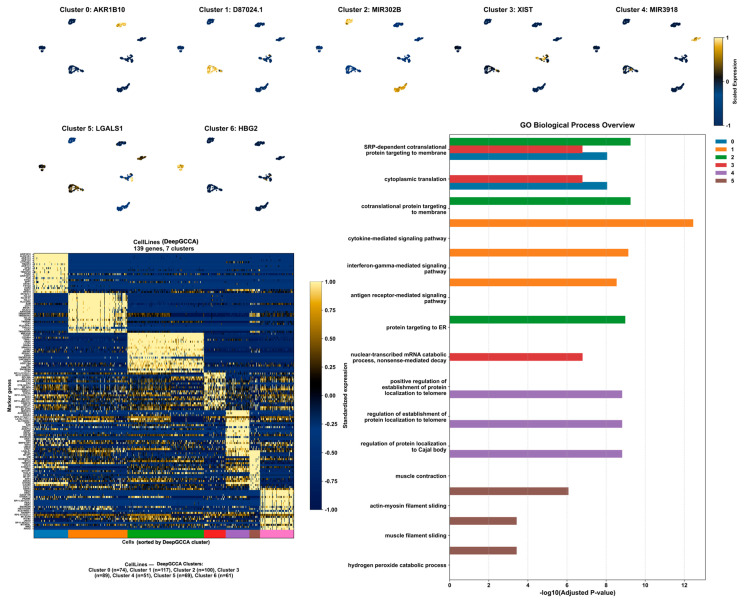
Cell-types heatmap and gene enrichment analysis of the CellLines dataset [33]. Top panels: UMAP projections showing the expression distribution of seven key marker genes across seven distinct cell clusters. Heatmap (**left**): Standardized expression of 139 marker genes across the identified cell clusters, highlighting distinct transcriptional modules. GO enrichment (**right**): Top biological processes enriched in each cluster, with bar lengths indicating the statistical significance (−log10(adjusted *p*-value)).

**Figure 11:**
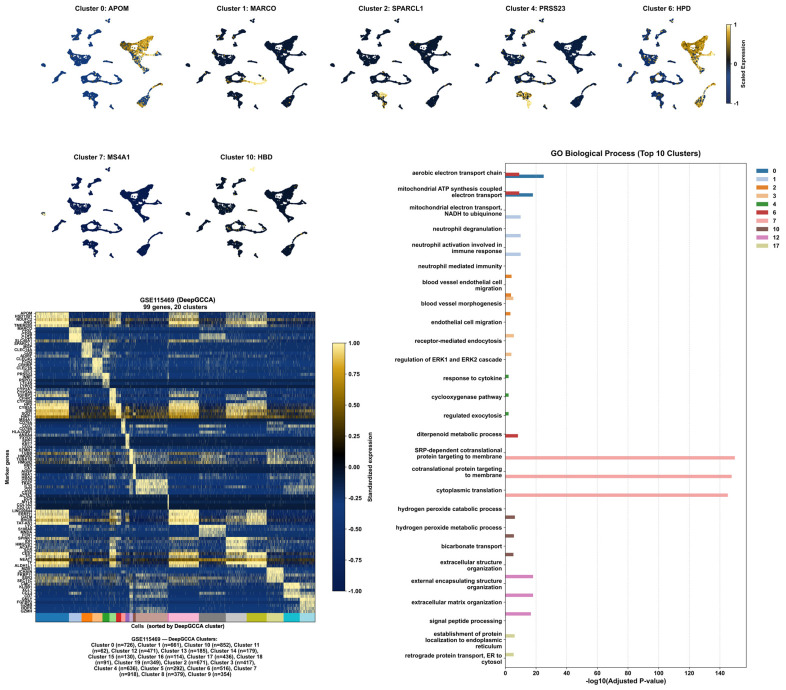
Cell-types heatmap and gene enrichment analysis of the GSE115469 dataset [34]. Top panels: UMAP projections showing the expression distribution of seven key marker genes across seven distinct cell clusters. Heatmap (**left**): Standardized expression of ninety-nine marker genes across the identified cell clusters, highlighting distinct transcriptional modules. GO enrichment (**right**): Top biological processes enriched in each cluster, with bar lengths indicating the statistical significance (−log10(adjusted *p*-value)).

After obtaining clustering labels from DeepGCCA, this study performed downstream biological interpretation on three authentic scRNA-seq datasets (Mouse [32], CellLines [33], GSE115469 [34]) in Figure 9, Figure 10 and Figure 11. These integrative analyses systematically uncover distinct biological landscapes across tissues and species: the Mouse dataset delineates the stepwise pancreatic endocrinogenesis from ductal progenitors to mature islets; the CellLines dataset reveals the functional heterogeneity and cytoskeleton-driven motility of various immune subpopulations; and the GSE115469 dataset dissects the metabolic, angiogenic, and immune coordination within the human liver microenvironment. To identify cluster-specific marker genes for downstream functional annotation and heatmap visualization, we employed the gene specificity score defined in the Supplementary Materials (Equation (S1)), which integrates group-specific expression advantage, absolute expression level, and overall variability to prioritize robust candidate markers. The top 10 key marker genes for each functionally important cell population, along with their assigned cell-type annotations, are provided in Tables S7–S9. The specific interpretations for each dataset are as follows:

In Figure 9, integrating the differential expression heatmap with Gene Ontology (GO) semantic enrichment, this study systematically reconstructs the dynamic differentiation trajectory of pancreatic endocrine cells from the ductal epithelium to mature islets in the Mouse dataset: Cluster 0 (Pancreatic Endocrine Progenitors) co-expresses ductal markers (*Spp1*, *Clu*, *Sparc*) alongside core developmental drivers (*Neurog3*, *Sox4*) [41], and is significantly enriched in pathways such as “SRP-dependent cotranslational protein targeting to membrane” and “protein targeting to ER (Endoplasmic Reticulum),” reflecting the active structural remodeling of cells physically detaching from the ductal epithelium and their requirement for robust basal translation to support early differentiation; subsequently, Cluster 2 (Fev+ Early Endocrine Lineage) represents an intermediate fate-determining state characterized by the relay expression of *Fev* and *Neurod1* following the downregulation of *Neurog3* [42], and is uniquely enriched in “regulation of mRNA splicing, via spliceosome” and “unfolded protein response (UPR),” precisely confirming that transitional cells require stringent post-transcriptional rearrangements and endoplasmic reticulum stress management to ensure the proper assembly of endocrine granules; finally, Cluster 1 (Mature Islet Endocrine Cells) specifically expresses hormone genes such as *Iapp*, *Pyy*, *Ins2*, and *Gcg* [41], and its robust enrichment in “regulation of insulin secretion” and “mitochondrial ATP (Adenosine Triphosphate) synthesis coupled electron transport” perfectly highlights the classic metabolism-secretion coupling phenotype of mature beta/alpha cells, which rely on mitochondrial oxidative phosphorylation to generate ATP for hormone vesicle exocytosis [43,44].

In Figure 10, integrating the differential expression heatmap with Gene Ontology (GO) semantic enrichment reveals the high transcriptional modularity and functional heterogeneity of the immune subpopulations within the CellLines dataset. For instance, Cluster 0 (Naive T cells) highly expresses the cytoskeletal genes *ACTB* and *TMSB4X* [45] and is significantly enriched in pathways such as “SRP (Signal Recognition Particle)-dependent cotranslational protein targeting to membrane” and “cytoplasmic translation,” reflecting the active ribosomal metabolism required to maintain basal homeostasis and rapid surface receptor turnover [46]; Cluster 1 (antigen-presenting cells), characterized by the high expression of *HLA-DRA* and *LYZ* [47], shows robust enrichment in the “interferon-gamma-mediated signaling pathway” and “antigen receptor-mediated signaling pathway,” validating the core immune surveillance function of myeloid cells in responding to microenvironmental stimuli via rapid kinase cascades [48]; meanwhile, Cluster 2 (Plasma cells), characterized by the expression of *MZB1* and *JCHAIN* alongside abundant immunoglobulin genes (*IGHG*, *IGKC*), perfectly defines mature plasma cells responsible for secreting antibodies [49]; furthermore, Cluster 5 (B cells) marked by *CD79A* [50] and Cluster 4 (Natural Killer/cytotoxic T cells) expressing cytotoxic molecules *GZMB* and PRF1 [51] are uniquely enriched in “muscle contraction” and “actin-myosin filament sliding,” a profile that, in non-muscle immune cells, reveals the mechanism of utilizing hyperactive cytoskeletal remodeling to drive chemotactic migration and immunological synapse formation [52]; finally, the enrichment of “nuclear-transcribed mRNA catabolic process, nonsense-mediated decay” in activated subsets like Cluster 3 (T cells) [53] confirms the physiological necessity of these cells in executing strict post-transcriptional quality control to maintain cellular homeostasis.

Integrating the differential expression heatmap with Gene Ontology (GO) semantic enrichment, this study elucidates the high transcriptional heterogeneity and functional phenotypes of the human liver microenvironment in the GSE115469 dataset of Figure 11. It is worth noting that due to the large number of clusters in this dataset, to ensure clarity and representativeness, the top spatial feature plots only display the top 7 clusters with the largest cell populations, the GO semantic enrichment analysis presents the top 10 most abundant clusters, and the heatmap selects exactly 5 top highly expressed marker genes for each cluster. Specifically, Cluster 0 (hepatocytes) highly expresses marker genes such as *APOM* and *TTR* [34] and is significantly enriched in GO pathways including “aerobic electron transport chain” and “mitochondrial ATP synthesis coupled electron transport,” perfectly reflecting the core role of hepatocytes as the body’s metabolic hub requiring intense oxidative phosphorylation and energy homeostasis [54]; Cluster 2 (endothelial cells), characterized by the specific expression of *SPARCL1* and *MGP* [34], shows robust enrichment in “blood vessel endothelial cell migration” and “blood vessel morphogenesis,” validating at the molecular level this subpopulation’s critical function in driving hepatic vascular remodeling and maintaining endothelial barrier integrity [55]; concurrently, Cluster 1 (Kupffer cells/macrophages) marked by high *MARCO* expression [34] exhibits strong pathway association with “neutrophil degranulation” and “neutrophil activation involved in immune response,” revealing the vital mechanism by which liver-resident macrophages sense microenvironmental stress and synergize with other myeloid cells to execute local immune surveillance and inflammatory regulation [56]; additionally, the enrichment of “SRP-dependent cotranslational protein targeting to membrane” and “extracellular matrix organization” in highly secretory subsets further highlights the physiological demands of extracellular niche remodeling and heavy protein synthesis within the liver ecosystem.

## 3. Discussion

This study proposes DeepGCCA, a deep multi-view subspace learning framework for clustering scRNA-seq data. By integrating complementary low-dimensional representations and learning a shared latent subspace under a weighted GCCA objective [30], DeepGCCA offers a robust strategy for multi-view fusion in unsupervised cell clustering. Consistent improvements were observed across both simulated and real datasets. Specifically, on two simulated datasets and three real scRNA-seq datasets, DeepGCCA achieved the best or highly competitive ARI/NMI scores relative to classical clustering baselines and widely used single-cell analysis pipelines. The performance gains were most pronounced on challenging real datasets, where technical noise and biological heterogeneity often induce substantial overlap in low-dimensional embeddings. This pattern suggests that cross-view correlation alignment, together with weighted fusion, can stabilize clustering outcomes under complex biological and technical variation. The qualitative visualization results further corroborate these quantitative findings. On the continuous-trajectory simulation (Sim1), DeepGCCA more faithfully preserves branch-level structure and local mixing patterns near transitional regions, indicating that the learned common subspace captures both global organization and local continuity. On the discrete simulation (Sim2), DeepGCCA maintains compact cluster geometry and well-defined boundaries while exhibiting more stable handling of outliers. Similar trends are evident in real datasets, where DeepGCCA generally produces more coherent and better separated clusters than traditional baselines, thereby providing a clearer structural basis for downstream biological interpretation.

To better understand which design choices drive these improvements, we conducted ablation experiments on the major loss components. The results indicate that reconstruction and GCCA-based alignment must be balanced within a moderate range: overly small weights weaken information retention or cross-view agreement, whereas overly large weights allow a single objective to dominate optimization and impair clustering. Entropy regularization is essential to prevent view-weight collapse and to promote balanced multi-view fusion. The superiority of DeepGCCA over single-view clustering further highlights the value of multi-view fusion. Different low-dimensional views capture complementary aspects of the data: one may emphasize global variance structure, another may better preserve neighborhood relationships, and another may highlight gradual transitions. Learning a shared subspace and performing weighted fusion enable DeepGCCA to leverage these complementary signals while suppressing view-specific noise, resulting in more accurate and robust clustering. In addition to performance, training stability is crucial for practical applicability. The monitored loss curves indicate smooth and stable optimization. Reconstruction loss decreases rapidly during early epochs, suggesting that dominant structures are learned quickly, whereas GCCA loss decreases more slowly, consistent with correlation alignment becoming effective once view-specific representations have stabilized [30]. The lack of strong oscillations further indicates reliable training dynamics for the proposed objective.

Despite these strengths, several limitations remain. First, the current implementation relies on a fixed set of representation views; incorporating more diverse or adaptive view-construction strategies may further improve robustness. Second, full-batch training can impose memory constraints on very large datasets, motivating the exploration of mini-batch or other scalable optimization schemes. Third, although key hyperparameters operate within a relatively stable range, some dataset-specific tuning may still be required to achieve optimal performance. Looking ahead, future work will focus on extending the framework to multi-omics settings by treating each modality as a view, improving scalability via mini-batch training and gradient accumulation, and developing adaptive mechanisms for hyperparameter selection and view-weight learning to reduce manual tuning.

Furthermore, the downstream marker gene analysis further confirms the biological validity of the clustering results. As shown in Figure 9, each cell subpopulation exhibits distinct and functionally coherent marker expression modules across the three real datasets. In the Mouse Pancreas data [32], differential expression of ribosomal protein genes and endocrine-related markers reflects variations in fundamental transcriptional activity among cell populations. In the CellLines data [33], the enrichment patterns of metabolic/detoxification genes and immune markers align with known heterogeneity in colorectal tumors. In the GSE115469 [34] liver dataset, modular upregulation of hepatocyte-secreted and immune-related genes is consistent with existing human liver cell atlases. These results collectively demonstrate that DeepGCCA captures biologically meaningful cell subpopulations at the molecular level, providing a solid foundation for downstream cell-type annotation and functional studies. In summary, DeepGCCA provides an effective and interpretable multi-view clustering solution for scRNA-seq data. Our results emphasize the importance of balancing reconstruction and cross-view alignment, the necessity of entropy-based regularization for stable fusion, and the practicality of enforcing orthogonality through hard constraints.

## 4. Materials and Methods

The DeepGCCA algorithm is a deep multi-view subspace representation learning method that integrates view encoding, maximum correlation subspace representation, and K-means clustering modules. This design effectively unifies the latent spaces across different views, improving the clarity of cell subpopulations. The specific steps are as follows.

### 4.1. Data Input and Preprocessing

Data preprocessing: Let the omics dataset be denoted as $X\in {\mathbb{R}}^{N\times G}$, where *N* denotes the number of cells and *G* denotes the number of genes; each element of ***X*** is denoted as $x_{ij}$ (representing the expression level of gene *j* in cell *i*). For RNA-seq datasets, first filter out mRNA molecules that are uniformly expressed across all cells and cells with valid gene expression counts below 200.

Next, the total expression counts of each cell were normalized using the CPM (Counts Per Million) method, which scales the total UMI (Unique Molecular Identifier) count per cell to 10^4^. This was followed by a ln(*x* + 1) transformation of the expression values. Finally, each gene (column) was standardized such that its mean was 0 and its variance was 1 [4].

(1) $${\tilde{x}}_{ij}=\frac{\log\left(\frac{x_{ij}}{\sum_{k=1}^{m}x_{ik}+\varepsilon }\times 10^{4}+1\right)-\mu_{j}}{\sigma_{j}}$$

Normalization eliminates variations in sequencing depth (i.e., total UMI count) between cells, prevents discrepancies caused by differences in the quantity of mRNA detected across different cells, and avoids errors resulting from division by zero. The logarithmic transformation, widely used in single-cell research, preserves zero values while compressing the dynamic range of high-expression values, enabling low-expression genes to participate in subsequent analyses and effectively preventing high-expression genes from dominating clustering results. Standardization sets the mean of each gene (or feature) to 0 and the standard deviation to 1 across different cells, eliminating absolute differences in gene expression levels and ensuring that all genes are treated equally by subsequent dimensionality reduction methods (PCA, UMAP). In methods such as Deep GCCA that rely on covariance matrices, normalization is an essential step, as CCA fundamentally analyzes correlations and requires standardized data.

### 4.2. Multi-View Generation

Building on the standardized data, we generate multiple distinct views to capture complementary data characteristics that a single representation might miss. Data dimensionality reduction: Multiple views are generated using various dimensionality reduction methods, including Principal Component Analysis (PCA), Uniform Manifold Approximation and Projection (UMAP), and Potential of Heat-diffusion for Affinity-based Trajectory Embedding (PHATE), among others. The dimensionality reduction formula for the *r*-th view is as follows:

(2) $$V^{\left(r\right)}=T_{r}(X)$$

Different dimensionality reduction views capture distinct features: PCA focuses primarily on the linear characteristics of cell data, while UMAP effectively represents its nonlinear features. All dimensionality reduction approaches compress raw gene expression data into a fixed 20-dimensional space. Each resulting view is formalized as an individual 20-dimensional feature vector.

### 4.3. Deep Autoencoder for View-Specific Encoding

Although these multi-view representations provide diverse perspectives, they may still contain noise and redundancies. To extract more robust, view-specific features, we independently process each view through a deep autoencoder. Weighted reconstruction multimodal autoencoder: Autoencoders effectively compress data dimensionality, removing noise while preserving essential biological information. Each view employs an independent two-layer neural network to generate a latent feature, which is then decoded to reconstruct the data. The encoding and decoding processes for the *r*-th view are detailed below.

(3) $$Z^{\left(r\right)}=f_{r}(V^{\left(r\right)})=\mathrm{LayerNorm}\left(\mathrm{ReLU}\left(V^{\left(r\right)}W_{r}+b_{r}\right)\right)$$

(4) $${\hat{V}}^{\left(r\right)}=f_{r}^{\prime }(Z^{\left(r\right)})=\mathrm{Sigmoid}\left(Z^{\left(r\right)}{W^{\prime }}_{r}+{b^{\prime }}_{r}\right)$$

Among these, the weight matrix **W** and the bias vector **b** determine the output of next layer; the LayerNorm() function performs independent normalization on each sample.

The reconstructed loss function ensures that the encoding process retains as much information as possible from the original view.

(5) $$L_{\mathrm{recon}}=\frac{1}{M}\sum_{r=1}^{M}L_{\mathrm{recon},r}=\frac{1}{M}\sum_{r=1}^{M}\frac{1}{nd_{r}}{\left\|{\hat{V}}^{\left(r\right)}-V^{\left(r\right)}\right\|}_{F}^{2}$$

### 4.4. Common Subspace Learning via Weighted GCCA

The autoencoder produces a set of low-dimensional latent representations, but these remain in separate feature spaces specific to each view. To enable cross-view comparison and subsequent fusion, we next align them into a shared common subspace. Maximum common subspace: Before solving for the maximum subspace, the encoded data must undergo centering and row L2 normalization. For the encoding matrix $Z^{\left(r\right)}\in {\mathbb{R}}^{N\times h}$ (where *h* denotes the encoding dimension), the transformation formula is as follows:

(6) $$H_{i,j}^{\left(r\right)}=\frac{Z_{i,j}^{\left(r\right)}-\frac{1}{N}\sum_{k=1}^{N}Z_{k,j}^{\left(r\right)}}{\sqrt{\sum_{j^{\prime }=1}^{h}{\left(Z_{i,j^{\prime }}^{\left(r\right)}-\frac{1}{N}\sum_{k=1}^{N}Z_{k,j^{\prime }}^{\left(r\right)}\right)}^{2}+\varepsilon }}$$

For each view, after encoding and applying centralization along with row L2 normalization to the resulting matrix, we introduce a learnable projection matrix $U^{\left(r\right)}\in {\mathbb{R}}^{h\times c}$ (where c denotes the dimension of the common subspace); require that $U^{\left(r\right)}$ satisfy orthogonality: $U^{(r)T}U^{\left(r\right)}=I_{c}$. The common subspace dimension *c* is set to 18, which is determined empirically via grid search and validation on clustering performance (ARI and NMI) using a held-out validation set. The representation projected onto the common subspace is given by $P^{\left(r\right)}=H^{\left(r\right)}U^{\left(r\right)}\in {\mathbb{R}}^{N\times c}$.

### 4.5. View-Weight Learning and Weighted Fusion

Once all views reside in the common subspace, their individual contributions to the final representation may still differ in quality and relevance. To adaptively balance these contributions, we introduce a view-weight learning mechanism. Obtain a set of view-weight parameters $\omega_{r}$, then normalize them using softmax. The calculation formula is

(7) $$w_{r}=\frac{\exp(\alpha_{r})}{\sum_{s=1}^{M}\exp(\alpha_{s})},\;r=1,\ldots ,M$$

The final fusion is represented as a weighted sum of the projections from each view.

(8) $$F=\sum_{r=1}^{M}w_{r}P^{\left(r\right)}$$

After training, the fused representation **F** is used as input to perform clustering using the K-means algorithm [9], where the number of clusters K is determined either by the actual number of categories (known) or by the elbow method.

### 4.6. The Loss Function

The entire architecture is trained end-to-end by simultaneously optimizing the autoencoder reconstruction, the weighted GCCA objective, and a view-weight regularization term. The weighted cross-covariance trace between views is calculated using the normalized $H^{\left(r\right)}$. First, define the covariance matrix between views *r* and *s* (*r* ≠ *s*) as $C_{rs}=\frac{1}{N}H^{(r)T}H^{\left(s\right)}\in {\mathbb{R}}^{h\times h}$, and the weighted cross-covariance sum (only for *r* ≠ *s*) as $C_{\mathrm{cross}}=\sum_{r=1}^{M}\sum_{\begin{array}{c} s=1\\ s\neq r \end{array}}^{M}w_{r}w_{s}C_{rs}$. The GCCA loss is then defined as

(9) $$L_{\mathrm{gcca}}=1-\frac{1}{h}tr(C_{\mathrm{cross}})$$

Without further regularization, the model may collapse to relying on a single view, ignoring the complementary information in others. An entropy regularization penalty term is introduced to evenly distribute view weights. It avoids single-view dominance and balances contributions from all views, defined as

(10) $$L_{\mathrm{entropy}}=-\sum_{r=1}^{M}w_{r}\log(w_{r}+\varepsilon )$$

The total loss function comprises three terms: reconstruction loss (Recon), GCCA loss (maximizing cross-view correlation), and entropy regularization (balancing view contributions). These three terms address different aspects of the learning process, and their relative importance is controlled by the hyperparameters *λ*_1_, *λ*_2_, and *λ*_3_, which can be adjusted based on validation performance. The normalization factor $S=\lambda_{1}+\lambda_{2}+\lambda_{3}$, and the total loss function is as follows:

(11) $$L_{\mathrm{total}}=\frac{\lambda_{1}}{S}L_{\mathrm{recon}}+\frac{\lambda_{2}}{S}L_{\mathrm{gcca}}+\frac{\lambda_{3}}{S}L_{\mathrm{entropy}}$$

### 4.7. Number of Cell Clusters

After jointly training the view encoder, projection matrix, and view weights, we obtained the final fusion representation **F** (Formula (8)). To categorize cells into distinct types, we adopted the K-means algorithm as the core clustering module. K-means offers low computational complexity, fast convergence and great scalability for large-scale datasets, and achieves promising performance when clusters are convexly separable in the feature space. The common subspace learned via DeepGCCA fuses complementary information across multiple views into a unified low-dimensional embedding, which generates compact, well-separated clusters for homogeneous cells. This characteristic naturally meets the basic assumptions of K-means, justifying its adoption for the final clustering procedure in our framework. For datasets with annotated ground-truth cell types, the cluster number K is directly assigned according to the real category count. This clustering operation enables downstream analysis and quantitative evaluation, and ultimately outputs definitive cell-type assignments.

### 4.8. Evaluation Metrics

For annotated datasets with ground-truth labels, we employ several metrics to evaluate clustering efficacy, including the Adjusted Rand Index (ARI) and Normalized Mutual Information (NMI). The Adjusted Rand Index (ARI) is derived from the Rand Index, which quantifies the similarity between two data partitions—namely, the ground-truth labels and the clustering results. To define ARI, we consider four counts based on pairwise comparisons of objects (cells): (1) *u*: the number of pairs that are placed together in both the true labeling A and the predicted labeling B. (2) *z*: the number of pairs that are separated into different groups in both A and B. (3) *w*: the number of pairs that belong to the same cluster in A but fall into different clusters in B. (4) *v*: the number of pairs that are assigned to different clusters in A but are grouped together in B.

(12) $$\mathrm{ARI}=\frac{C_{z}^{2}(u+v)-\left[\left(u+z)(u+w)+(w+v)(z+v\right)\right]}{C_{z}^{2}-\left[\left(u+z)(u+w)+(w+v)(z+v\right)\right]}$$

NMI is defined as the mutual information between *P* and *Q* normalized by their entropies.

(13) $$H(P)=-\sum_{m=1}^{M}|P_{m}|\log\frac{\left|P_{m}\right|}{N}$$

(14) $$I(P,Q)=\sum_{m=1}^{M}\sum_{g=1}^{G}|P_{m}\cap Q_{g}|\log\frac{n|P_{m}\cap Q_{g}|}{\left|P_{m}|\times |Q_{g}\right|}$$

(15) $$NMI(P,Q)=\frac{2I(P,Q)}{H(P)+H(Q)}$$

where *P* and *Q* denote two partition results of the same dataset, *P* usually represents the clustering result and *Q* represents the ground-truth labels or the reference partition. *N* denotes the total number of samples. *M* and *G* denote the numbers of clusters in *P* and *Q*, respectively. $P_{m}$ denotes the sample set of the *m*-th cluster in partition *P*, and $Q_{g}$ denotes the sample set of the *g*-th cluster in partition *Q*. $\left|P_{m}\right|$ and $\left|Q_{g}\right|$ denote the numbers of samples contained in the corresponding clusters, while $\left|P_{m}\cap Q_{g}\right|$ denotes the number of samples shared by both clusters. H(P) and H(Q) denote the entropies of partitions *P* and *Q*, respectively, and I(P,Q) denotes the mutual information between the two partitions.

### 4.9. Parameter Setting

This section details the numerical configurations governing the algorithmic logic to ensure the rigorous reproducibility of the DeepGCCA framework and facilitate its application to diverse transcriptomic data. The systematic establishment of hyperparameters relies on both theoretical constraints and empirical optimizations across various developmental topologies. Table 5 provides a comprehensive summary of the specific parameters used for Preprocessing, View Generation, DeepGCCA, Loss Weights and Clustering.

The DeepGCCA model adopts an end-to-end joint training strategy, in which all trainable parameters (including view encoders, decoders, projection matrices $U^{\left(r\right)}$, view-weight parameters $\alpha_{r}$, and temperature coefficients τ) are optimized by minimizing the total loss function. During training, the entire dataset is fed into the model as a single batch (i.e., full-batch gradient descent), with the batch size equal to the total number of cells. Optimization Algorithm and Learning Rate: The Adam optimizer is utilized with an initial learning rate of 1 × 10^−3^. To accelerate convergence and avoid oscillation, a learning rate decay strategy is applied: If the total loss does not decrease for 10 consecutive epochs, the learning rate is multiplied by a decay factor of 0.5. In addition, gradient clipping is set with a maximum norm of 1.0 to prevent gradient explosion.

Training Termination Condition: The model is trained for a maximum of 200 epochs. If the total loss drops below a predefined threshold of 0.1, training is stopped early, and the model parameters of the current epoch are saved. Otherwise, the parameters corresponding to the lowest training loss are retained as the final model. Enforcement of Orthogonal Constraints: To strictly ensure that the projection matrix $U^{\left(r\right)}$ satisfies the orthogonality condition $U^{(r)T}U^{\left(r\right)}=I_{c}$, we explicitly project each $U^{\left(r\right)}$ onto the Stiefel manifold via QR decomposition after every forward propagation step. Because this hard constraint directly enforces orthogonality, we no longer incorporate an orthogonal loss. View Weights and Temperature Coefficient: The view weights are normalized using the softmax function. The temperature coefficient τ is initialized to 1 and can be adaptively updated during training; in this work, it is fixed at 1 for simplicity of analysis. Hyperparameter settings: The weights of each component in the total loss function are set as follows according to the implementation: *λ*_1_ = 1, *λ*_2_ = 0.1, and *λ*_3_ = 0.1. The latent space dimension h is set to 64, and the common subspace dimension c is set to 18. All hyperparameters were initially optimized based on clustering performance on the validation set and then fixed for all experiments. Hardware and Framework: All experiments were implemented in Python 3.9.25 using PyTorch 2.5.1 and were conducted on a Windows-based CPU-only workstation with 8 physical cores, 16 logical threads.

## 5. Conclusions

This work presents DeepGCCA, a deep multi-view clustering framework that addresses the high dimensionality, sparsity, and noise of scRNA-seq data by fusing complementary molecular views through a weighted GCCA objective and performing clustering in a unified common subspace. Across simulated and real datasets, DeepGCCA achieves superior or highly competitive clustering accuracy (ARI and NMI), with qualitative embeddings confirming improved cluster coherence and separability. Ablation studies reveal that a balanced trade-off between reconstruction and GCCA alignment is critical, while entropy regularization prevents view-weight collapse and stabilizes multi-view fusion. Comparisons with single-view baselines confirm that multi-view integration yields more informative representations, and the smooth training dynamics support the practical reliability of the method. Cluster-specific marker-gene heatmaps further validate the transcriptomic interpretability of the identified cell populations.

Nevertheless, several limitations must be acknowledged. The current evaluation assumes knowledge of the true number of clusters K, and automatic cluster-number estimation remains an open direction for future fully unsupervised applications. Broader validation on more diverse real datasets—with varied batch structures, sparsity levels, and rare cell types—is also deferred to subsequent work. Beyond these immediate extensions, the framework is adaptable to other single-cell modalities and multi-omics data, and its design offers general insights into loss balancing and constraint enforcement for deep multi-view learning. Within the scope of this study, DeepGCCA provides an efficient, robust, and interpretable solution for unsupervised single-cell transcriptomic clustering, with a clear roadmap for addressing its current limitations in future work.

## Figures and Tables

**Table 1:** Summary of datasets.

Dataset	Cells	Genes	Cluster Number	Description	Source
Sim1	1000	1800	4	Continuous	[31]
Sim2	2000	2500	3	Discrete	[31]
Mouse	2780	27,990	3	Real	[32]
CellLines	561	57,241	7	Real	[33]
GSE115469	8439	20,007	20	Real	[34]

**Table 2:** Average of ARI for six methods of five datasets.

Method	Sim1	Sim2	Mouse	CellLines	GSE115469
K-means	0.625 ± 0.05	0.981 ± 0.064	0.563 ± 0.0241	0.635 ± 0.146	0.508 ± 0.037
Spectral	0.585 ± 0.00	1 ± 0	0.581 ± 0.000	0.663 ± 0.006	0.4094 ± 0.001
scMDC	0.536 ± 0.043	0.927 ± 0.117	0.524 ± 0.0147	0.657 ± 0.051	0.3805 ± 0.030
Seurat	0.408 ± 0	1 ± 0	0.327 ± 0	0.7886 ± 0	0.6009 ± 0
scMMN	0.354 ± 0.10	0.8883 ± 0.207	0.210 ± 0.123	0.615 ± 0.08	0.3779 ± 0.0376
DeepGCCA	0.719 ± 0.11	1 ± 0	0.613 ± 0.125	0.650 ± 0.06	0.562 ± 0.030
DeepGCCA best	0.8726	1	0.8559	0.6858	0.6336

**Table 3:** Average of NMI for six methods of five datasets.

Method	Sim1	Sim2	Mouse	CellLines	GSE115469
K-means	0.698 ± 0.0234	0.982 ± 0.058	0.577 ± 0.017	0.736 ± 0.067	0.706 ± 0.017
Spectral	0.676 ± 0.004	1 ± 0	0.580 ± 0.000	0.773 ± 0.002	0.684 ± 0.000
scMDC	0.665 ± 0.021	0.934 ± 0.103	0.561 ± 0.014	0.779 ± 0.033	0.654 ± 0.018
Seurat	0.525 ± 0	1 ± 0	0.3897 ± 0	0.836 ± 0	0.7133 ± 0
scMMN	0.437 ± 0.10	0.891 ± 0.193	0.208 ± 0.107	0.763 ± 0.063	0.6510 ± 0.0261
DeepGCCA	0.748 ± 0.09	1 ± 0	0.605 ± 0.082	0.770 ± 0.039	0.7442 ± 0.017
DeepGCCA best	0.870	1	0.7936	0.7972	0.7653

**Table 4:** Ablation study of loss components of Mouse dataset in DeepGCCA.

Loss Term	Weight	ARI	NMI
Recon	0	0.6581 ± 0.0459	0.7045 ± 0.0680
	0.01	0.6548 ± 0.0503	0.7112 ± 0.0541
	0.1	0.6184 ± 0.0885	0.6359 ± 0.1336
	1	0.6812 ± 0.0661	0.7341 ± 0.0730
	5	0.6817 ± 0.0649	0.7290 ± 0.0687
GCCA	0	0.6449 ± 0.0768	0.6632 ± 0.1233
	0.01	0.6812 ± 0.0661	0.7341 ± 0.0730
	0.1	0.6619 ± 0.0842	0.6970 ± 0.1071
	1	0.6194 ± 0.0888	0.6374 ± 0.1343
	5	0.6070 ± 0.0711	0.6235 ± 0.1141
Entropy	0	0.6648 ± 0.0727	0.6974 ± 0.1106
	0.01	0.6695 ± 0.0777	0.7141 ± 0.0979
	0.1	0.6812 ± 0.0661	0.7341 ± 0.0730
	1	0.6643 ± 0.0837	0.7001 ± 0.1161
	5	0.6380 ± 0.0867	0.6566 ± 0.1230

**Table 5:** Summary of hyperparameters and parameter configurations in DeepGCCA.

Module	Parameter	Symbol	Value
Preprocessing	Low-quality cell filtering	n_expr_	≥200 expressed genes
	Library-size normalization	L_i_	10^4^ per cell
	Log transformation	f(x)	ln(x + 1)
	Highly variable gene selection	N_HVG_	2000
	Gene-wise standardization	μ_j_, σ_j_	Zero mean, unit variance
View Generation	View-generation seed	VIEW_SEED	42
	PCA/UMAP/PHATE views	*d*	20
	UMAP/PHATE settings	n_neighbors_, min_dist_, knn	15, 0.3, 10
DeepGCCA	Encoder hidden dimension	*h*	64
	Common subspace dimension	*c*	18
	Maximum epochs	E	200
	Loss threshold	T_loss_	0.1
	Learning rate	lr	0.001
Loss Weights	Reconstruction loss weight	*λ*_1_	[0.1~1]
	GCCA loss weight	*λ*_2_	[0.1~1]
	Entropy regularization weight	*λ*_3_	[0~0.1]
Clustering	Clustering method	KMeans:max_iter	300
	Cluster number	K	True number of classes
	Random seed	seed	0, 1, 6, 15, 19, 23, 32, 48, 49, 54, 61, 66, 68, 73, 78, 85, 89, 91, 96, 100

## Data Availability

The source code for DeepGCCA is openly available on the GitHub repository at https://github.com/huaizhu-suns/DeepGCCA (accessed on 22 June 2026). The simulated datasets (Sim1 and Sim2) were generated using the scMultiSim simulator [31]. The Mouse Pancreas dataset is available from the corresponding author of Long et al. (2026) upon reasonable request [32]. The human colorectal tumor CellLines dataset is available from Li et al. (2017) (PMID: 28319088) [33]. The GSE115469 dataset is publicly available from the Gene Expression Omnibus [34]. All other data used in this study are included in the manuscript or can be obtained from the original publications cited.

## Supplementary Materials

The following supporting information can be downloaded at: https://www.mdpi.com/article/10.3390/ijms27135819/s1.

## Abbreviations

The following abbreviations are used in this manuscript:

scRNA-seq	single-cell RNA sequencing
RNA-seq	RNA sequencing
PCA	principal component analysis
UMAP	uniform manifold approximation and projection
PHATE	Potential of Heat-diffusion for Affinity-based Trajectory Embedding
k-NN	k-nearest neighbor
DeepGCCA	deep generalized canonical correlation analysis
GCCA	generalized canonical correlation analysis
CPM	Counts Per Million
UMI	Unique Molecular Identifier (or Unique Molecular Index)
ARI	Adjusted Rand Index
NMI	Normalized Mutual Information
scMDC	Clustering of single-cell multi-omics data with a multimodal deep learning method
scMMN	Multi-level multi-view network based on structural contrastive learning for scRNA-seq data clustering
