# Supplementary material for: Integrating Multi-View Features via Deep Generalized Canonical Correlation Analysis for Single-Cell Clustering
Source: Int J Mol Sci. 2026 Jun 27;27(13):5819. doi: 10.3390/ijms27135819 (PMC13361346; doi:10.3390/ijms27135819)
Supplement: Supplementary file 1 [file ijms-27-05819-s001.zip › ijms-4342653-supplementary.pdf]

Supplementary Materials

The gene specificity score was defined as in Equation S1:

$$\text{Score}_{ij} = \left(\text{Mean}_{ij} - \max_{k \neq i}\left(\text{Mean}_{kj}\right)\right) + 0.15\text{Mean}_{ij} + 0.1\text{Var}_{j} \quad \text{(S1)}$$

where Mean_{ij} is the mean expression of gene *j* in group *i*, $\max_{k \neq i}\left(\text{Mean}_{kj}\right)$ is the maximum mean expression of the same gene across all other groups, and Var_{j} is the overall variance of gene *j*. The first term quantifies group specificity by measuring the expression advantage of gene *j* in the target group over the most competitive non-target group. The second term weights genes with higher absolute expression, while the third term favors genes with greater overall variability. Together, this composite score prioritizes genes that are specifically enriched, abundantly expressed, and highly variable, thereby facilitating the identification of candidate marker genes.

Table S1. Paired statistical tests against the strongest baseline on each dataset for ARI

Dataset	Best Baseline	DeepGCCA (mean±std)	Baseline (mean±std)	p-value (ARI)
Sim1	K-means	0.704±0.123	0.625±0.054	<0.05
Sim2	Seurat / Spectral	1.000±0	1.000±0	N/A (saturated)
Mouse	Spectral	0.651±0.123	0.581±0.000	<0.05
Cell	Seurat	0.652±0.049	0.789±0	<0.05
GSE	Seurat	0.521±0.031	0.601±0	<0.05

Table S2. Paired statistical tests against the strongest baseline on each dataset for NMI

Dataset	Best Baseline	DeepGCCA (mean±std)	Baseline (mean±std)	p-value (NMI)
Sim1	K-means	0.732±0.131	0.698±0.024	>0.05
Sim2	Seurat / Spectral	1.000±0	1.000±0	N/A (saturated)
Mouse	Spectral	0.634±0.080	0.580±0.001	<0.05
Cell	Seurat	0.780±0.032	0.836±0	<0.05
GSE	K-means	0.726±0.015	0.716±0.017	>0.05

Table S3. Baseline-tuning table for six methods

Method	Software Version	Preprocessing	Key Parameters
DeepGCCA	Python 3.9.25; numpy 1.26.4; pandas 2.3.3; scikit-learn 1.5.1; torch 2.5.1;umap-learn 0.5.3;phate 2.0.0	Filter low-quality cells (cells with <200 expressed genes); library-size normalization to 1e4 per cell; ln(x+1) transformation; select the top 2000 highly variable genes by gene-wise variance; gene-wise standardization; removal of constant genes	TARGET_DIM=20, DGCCA_HIDDEN_DIM=64, DGCCA_COMMON_DIM=18, DGCCA_EPOCHS=200, DGCCA_LR=1e-3(PCA)n_neighbors=15, min_dist=0.3, n_components=TARGET_DIM, TARGET_DIM=20(umap);knn=10, t='auto', n_components=TARGET_DIM, TARGET_DIM=20(phate)
KMeans	scikit-learn 1.5.1	Filter low-quality cells (cells with <200 expressed genes); library-size normalization to 1e4 per cell; ln(x+1) transformation; select the top 2000 highly variable genes by gene-wise variance; gene-wise standardization; removal of constant genes	n_init='auto', max_iter=300, random_state=66
Spectral	scikit-learn 1.5.1	Filter low-quality cells (cells with <200 expressed genes); library-size normalization to 1e4 per cell; ln(x+1) transformation; select the top 2000 highly variable genes by gene-wise variance; gene-wise standardization; removal of constant genes	affinity='rbf', gamma=1.0 / X_scaled.shape[1], n_init=10, random_state=50
scMDC	Python 3.9.25; torch 2.5.1; scanpy 1.10.3; scikit-learn 1.5.1	Filter low-quality cells (cells with <200 expressed genes); select the top 2000 highly variable genes by gene-wise variance; apply size-factor normalization, input normalization, and	encodeLayer=[256,64,32,16], decodeLayer1=[16,64,256], decodeLayer2=[16,64], activation='elu', sigma1=2.5, sigma2=1.5, gamma=0.1, tau=1.0, phi1=0.001, phi2=0.001, cutoff=0.5, batch_size=256, pretrain_epochs=300,

		log transformation	maxiter=1000, lr=0.1
scMMN	custom implementation; Python 3.9.25; torch 2.5.1; scikit-learn 1.5.1	Select the top 2000 highly variable genes by gene-wise variance; apply log1p transformation; standardize with StandardScaler; construct cosine- and Euclidean-distance KNN graphs; apply symmetric graph normalization and Laplacian smoothing	k=30, hidden_dim=256, out_dim=n_clusters, epochs=300, lr=0.01, alpha=1.0, beta=0.001, gamma=0.5
Seurat	R 4.5.2; Seurat 5.5.0; mclust 6.1.2; aricode 1.0.3; dplyr 1.1.4; anndata 0.8.0	Normalize the count matrix; select 2000 highly variable genes; scale the data; run PCA; construct the nearest-neighbor graph; cluster with resolution=0.5	FindVariableFeatures(nfeatures=2000), RunPCA(npcs=50), FindNeighbors(dims=1:30), FindClusters(resolution=0.5)

Table S4. Comparison of running times for 6 methods(s)

**Method**	**Sim1**	**Sim2**	**Mouse**	**CellLines**	**GSE115469**
K-means	0.1333	0.0567	0.1034	0.0405	1.045
Spectral	0.2621	0.2922	0.5882	0.0763	4.7191
scMDC	166.5758	358.0540	192.9523	42.8744	592.3673
Seurat	3.32	6.43	11.5	32.91	169.74
scMMN	98	397	414	22	3423.8
DeepGCCA	33	32	41	17	95

Table S5. Comparison of Memory for 6 methods(MB)

**Method**	**Sim1**	**Sim2**	**Mouse**	**CellLines**	**GSE115469**
K-means	353.48	410.65	1799.63	893.08	3544.5
Spectral	384.7	534.78	1829.38	929.89	3573.12
scMDC	662.88	863.49	2564.52	1736.04	5600.71
Seurat	2388.38	2933.34	4154.2	4033.34	10108.18
scMMN	765.45	1685.49	3079.50	1850.45	19134.75
DeepGCCA	746.18	929.56	2427.92	1277.04	3989.33

Table S6. Ablation study of loss components of Sim1 dataset in DeepGCCA.

Loss Term	Weight	ARI	NMI
	0	0.6886±0.1406	0.7597±0.0807
	0.01	0.7970±0.1382	0.7993±0.0869
Recon	0.1	0.7707±0.1369	0.8160±0.0981
	1	0.7641±0.1055	0.7933±0.0755
	5	0.7859±0.1136	0.8116±0.0787
	0	0.5540±0.1514	0.6568±0.0954
	0.01	0.7749±0.1223	0.8005±0.0894
GCCA	0.1	0.7641±0.1055	0.7933±0.0755
	1	0.7968±0.1373	0.8159±0.0983
	5	0.7053±0.1716	0.7701±0.1022
	0	0.7892±0.1231	0.8141±0.0866
	0.01	0.7815±0.1361	0.8105±0.0903
Entropy	0.1	0.7641±0.1055	0.7933±0.0755
	1	0.7545±0.1324	0.7867±0.0896
	5	0.6479±0.1729	0.7222±0.1196

Table S7. The top 10 key marker genes for each functionally important cell population of Mouse dataset.

Cluster ID	Top 10 Markers	Known Cell-type Annotation	References
Cluster 0	*Spp1, Clu, Sparc, Mdk, Neurog3, Sox4, Tmsb4x, Ifitm2, H19, Gadd45a*	Pancreatic Endocrine Progenitors	[41]
Cluster 1	*Iapp, Pyy, Rbp4, Ins2, Ttr, Tmem27, Gcg, Pcsk2, Slc38a5, Manf*	Mature Islet Endocrine Cells	[41]
Cluster 2	*Fev, Cck, Chgb, Chga, Cldn4, Neurod1, Tm4sf4, Krt7, Malat1, Runx1t1*	Fev+ Early Endocrine Lineage	[42]

Table S8. The top 10 key marker genes for each functionally important cell population of CellLines dataset.

Cluster ID	Top 10 Markers	Known Cell-type Annotation	References
Cluster 0	*EEF1A1, B2M, GAPDH, ACTB, TMSB4X, RPLP1, RPS18, RPL13, RPL8, RPS12*	Naive T Cells	[45]
Cluster 1	*CD74, HLA-DRA, HLA-DPB1, HLA-DPA1, HLA-DRB1, LYZ, SRGN, TYROBP, CTSS, FCER1G*	Antigen-Presenting Cells (APCs) / Dendritic Cells	[47]
Cluster 2	*IGKC, IGHG1, IGHG3, IGLC2, IGHG4, IGHM, IGHA1, JCHAIN, MZB1, IGLC3*	Plasma Cells / Plasmablasts	[49]
Cluster 3	*CD3D, IL32, TRBC2, LCK, CD3E, GZMA, CCL5, NKG7, TRAC, TRBC1*	T Cells	[53]
Cluster 4	*GNLY, NKG7, GZMB, PRF1, KLRB1, FGFBP2, KLRD1, GZMA, CST7, SPON2*	Natural Killer (NK) / Cytotoxic T Cells	[51]
Cluster 5	*MS4A1, CD79A, HLA-DRA, CD74, HLA-DPB1, HLA-DRB1, CD79B, HLA-DQA1, HLA-DQB1, BANK1*	B Cells	[50]
Cluster 6	*CD14, S100A9, S100A8, LYZ, FCN1, VCAN, TYROBP, CTSS, MNDA, S100A12*	CD14+ Monocytes	[47]

Table S9. The top 10 key marker genes for each functionally important cell population of GSE115469 dataset.

Cluster ID	Top Markers (10 Genes)	Known Cell-type Annotations	References
Cluster 0	*APOM, HSD11B1, NDUFC2, ANG, TMEM205, TTR, NDUFB9, MGST2, HSD17B10, NDUFB11*	Hepatocytes	[34,54]
Cluster 1	*MARCO, CD5L, C1QB, C1QA, SLC40A1, VCAM1, C1QC, MS4A7, CREG1, LIPA*	Kupffer Cells / Macrophages	[34,56]
Cluster 2	*SPARCL1, MGP, CLEC14A, CD9, ADIRF, TM4SF1, SLCO2A1, CAV1, EPAS1, FKBP1A*	Endothelial Cells	[34,55]
Cluster 7	*MS4A1, CD79A, CD37, CD79B, HLA-DQB1, CD52, LTB, LINC00926, IGHD, VPREB3*	B Cells	[34]
Cluster 10	*HBD, CA1, AHSP, HBA1, HBA2, ALAS2, HBM, HBB, SNCA, SLC4A1*	Erythrocytes	[34]
